# Deliberate practice in the age of artificial intelligence: how AI-assisted practice supports self-regulated learning and expert performance development in conservatory musicians

**DOI:** 10.3389/fpsyg.2026.1929520

**Published:** 2026-09-15

**Authors:** Lina Song

**Affiliations:** Public Arts Education and Teaching Center, Zhongyuan Institute of Science and Technology, Zhengzhou, China

**Keywords:** artificial intelligence, conservatory musicians, cross-lagged panel model, deliberate practice, expert performance, longitudinal mediation, self-regulated learning

## Abstract

**Introduction:**

Deliberate practice is central to expert performance development, yet the conditions under which practice produces sustained gains remain contested. AI-assisted music practice applications provide adaptive feedback, intelligent scheduling, automated error detection, and performance analytics, potentially supporting deliberate practice. This study examined whether engagement with AI-assisted practice applications is longitudinally associated with expert performance development among conservatory musicians through self-regulated learning (SRL).

**Methods:**

A three-wave longitudinal study was conducted over a 16-week academic semester with a target sample of 600 conservatory musicians. Of these, 512 completed all three waves (85.3%). Temporal associations among AI-assisted practice engagement, SRL, and instructor-rated musical performance were examined.

**Results:**

Significant longitudinal associations supported the proposed pathway. Greater engagement with AI-assisted practice applications was associated with subsequent SRL, which was in turn associated with later instructor-rated musical performance. These findings are consistent with a mediating role of SRL in the association between AI-assisted practice engagement and expert performance development.

**Discussion:**

AI-assisted practice applications may support expert performance development by fostering self-regulatory processes conducive to deliberate practice. However, because the study was observational rather than randomized and involved heterogeneous AI applications rather than a standardized intervention, the findings should be interpreted as longitudinal associations rather than causal effects. AI-assisted practice applications are therefore best conceptualized as pedagogical supports that facilitate conditions for deliberate practice rather than as deliberate practice itself.

## 1 Introduction

The artificial intelligence (AI) is increasingly being incorporated into music education and independent practice, creating new possibilities for supporting musicians; technical and expressive development. Conservatory students can now use AI-assisted practice applications that analyze performance, provide adaptive feedback, identify errors, monitor progress, and recommend targeted practice activities. These technologies extent feedback beyond formal instructional sessions and may provide learners with additional opportunities to monitor and regulate their practice. However, the extent to which engagement with AI-assisted practice applications are associated with sustained performance development, and the psychological mechanisms underlying such associations, remain insufficiently understood. These AI-assisted practice programs typically incorporate features like pitch and rhythm analysis, performance tracking, adaptive feedback, error detection, and personalized practice recommendations. They allow students to receive immediate formative feedback in between official classes.

Deliberate practice is a central concept in research on expertise development and has substantially influenced the study of performance acquisition since its formalization. It continues to be the subject of considerable scholarly interest, as well as debate and criticism. Hambrick et al. (2020) have recently provided a sober evaluation of the deliberate practice view, which suggests that practice quality does matter, but that the notion of deliberate practice as a singular causal factor is strongly qualified. Macnamara and Maitra (2019) revisited the original Ericsson et al. (1993) framework and questioned the extent to which its empirical foundations generalize consistently across performance domains. Harwell and Southwick (2021) further identified several enduring misconceptions surrounding the expert-performance approach, including interpretations that have become increasingly prominent with the emergence of new practice technologies and popularized claims concerning extensive practice hours. Importantly, emerging artificial intelligence (AI) technologies, although they may support several of the conditions associated with deliberate practice, should not be seen as substitutes for the structured effortful practice and expert supervision that are key to Ericsson’s approach.

In this study, AI-assisted deliberate practice refers to students’ engagement with various AI-supported music practice applications during individual practice sessions. These applications are not considered deliberate practice; rather, they are conceptualizing as pedagogical support tools that may create conditions conducive to deliberate practice. Typical provide automated pitch and rhythm analysis, tempo tracking, adaptive error detection, performance statistics, individualized practice recommendations and intelligent practice scheduling. These functions may support self-monitoring, goal setting, targeted repetition and reflection, but their presence does not by itself establish that deliberate practice has occurred. Accordingly. AI-assisted applications are conceptualized as complements to, rather than substitutes for, experienced teacher guidance.

None of this important literature, however, predicted the coming of AI-enabled practice tools that alter the feedback structure for deliberate practice. AI-powered music practice applications have changed the independent music practice landscape by offering instant, data-driven feedback that complements, rather than replaces, traditional teacher-led instruction. Unlike traditional practice environments, these technologies are continuously analyzing musical performances and providing learners with immediate feedback on aspects of performance such as pitch accuracy, rhythmic accuracy, tempo stability, articulation, intonation and expressive control. Also, AI-supported systems can offer personalized suggestions for practice, monitor learning progress, identify typical performance errors, and help the students in planning and regulating their practice sessions with adaptive feedback and intelligent practice scheduling (Li and Wang, 2024; Ou et al., 2025). Such functions strongly correspond to the self-monitoring, goal-setting, strategic adjustment, and reflective processes typical of self-regulated learning (Panadero, 2017). Thus, AI-assisted practice applications should be viewed as pedagogical technologies that facilitate the conditions required for deliberate practice by improving learners’ self-regulatory processes, not as substitutes for expert instruction or deliberate practice itself. This conceptual distinction provides the theoretical foundation for exploring how AI-supported practice environments could contribute to the development of expert performance in conservatory musicians. Accordingly, in the present study, AI-assisted practice refers to students’ engagement with AI-assisted music practice applications that provide adaptive feedback, performance monitoring, automated error detection, and personalized practice support during autonomous practice sessions. These applications are conceptualized as pedagogical tools that facilitate learners’ self-monitoring, goal setting and reflective practice, thereby strengthening the conditions necessary for deliberate practice without replacing expert instruction. Classical approaches to deliberate practice emphasize the importance of timely, targeted, expert feedback, which has traditionally been provided through individualized instruction and supervision by a teacher or coach (Ericsson et al., 1993). Ou et al. (2025) examined the role of AI-based practice applications in providing aspects of performance feedback and reported meaningful associations with self-efficacy and self-regulated learning capacity. Li and Wang (2024) examined the broader role of AI in music education and identified SRL as a potentially important but insufficiently theorized mechanism through which AI-supported learning tools may influence skill development.

Self-regulated learning (SRL) provides the primary psychological framework for this study. Taking a broader look at six influential models of SRL, Panadero (2017) identified a common emphasis on cyclical learning processes involving goal setting, monitoring, and reflection. These processes are also supported by several functions of AI-assisted music practice applications, such as progress monitoring, adaptive feedback, and practice planning. Tinajero et al. (2024) further noted that contemporary SRL models increasingly conceptualize self-regulation as a multi-dimensional and componential process that can be assessed at a finer level of detailed. This perspective is particularly relevant to the present three waves longitudinal design because it allows changes and temporal associations in SRL to be examined across the academic semester.

Does deliberate practice supported by AI-assisted music practice applications contribute to expert performance development by strengthening students’ SRL? To address this question, the present study examines instructor-rated musical performance across three waves conducted over a 16-week academic semester between conservatory musicians from different institutions and instrument families. Rather than relying on self-reported performance outcomes the study uses performance ratings provided by qualified instructors. The three-wave cross lagged panel analysis also examines the directionality of the longitudinal association between AI-assisted practice engagement and SRL or whether students with stronger initial SRL are more likely to engage with AI-assisted practice technologies.

The remainder of this article is organized as below. Section 2 presents the theoretical and empirical literature for the relevant domains of deliberate practice, self-regulated learning, AI-supported music learning, and music assessment. The three-wave longitudinal method is described, along with the sample characteristics, measurement instruments and analytic sequence, in Section 3. The results of the measurement model, the structural model, the mediation model and the cross-lagged panel model are presented in Section 4. The theoretical and practical implications of these findings as well as limitations are discussed in Section 5. Section 6 concludes.

## 2 Literature review

### 2.1 Deliberate practice: theoretical foundations and contemporary debates

Deliberate practice theory has been in an ambivalent position in expertise research, being at the same time one of the most cited theoretical frameworks in the psychology of skill acquisition, and one of the most contested. Hambrick et al. (2020) synthesized evidence from domains to determine that deliberate practice accounts for a meaningful but not comprehensive portion of variance in expert performance, along with significant roles for individual differences with respect to other capacities (e.g., working memory, domain-specific aptitudes, and starting age). Thus, a more measured reading of this has displaced earlier more absolutist pronouncements. It is possible to provide conceptual space for the discussion of mediating and moderating processes such as self-regulated learning that could explain why deliberate practice has more of an effect for some students than others.

Here, the reanalysis from Macnamara and Maitra (2019) is relevant as it returns the focus to the original theoretical structure of deliberate practice—practice that is effortful, explicit to a weakness, instructive and repeated with improvement. Several of these characteristics can be supported by contemporary AI-assisted music practice applications, which are designed to facilitate specific aspects of the deliberate practice process rather than replace it. For example, AI-assisted practice applications can support learners by identifying technical weaknesses through adaptive error detection, providing timely feedback using performance analytics, and encouraging structured repetition through intelligent practice scheduling. These functions are intended to complement learners’ effortful practice and teacher guidance rather than substitute for them. The extent to which these functions translate into improved performance remains an empirical question, but one that merits careful scrutiny of the self-regulated learning pathway, as that is the path by which learners will transform the feedback, human or artificial, that they have available into better performance.

There are some methodological templates offered by deliberate practice in applications specific to the domain, such as in clinical and applied contexts. In their study of deliberate practice training for delivering empathetic interventions, Tomás et al. (2025) showed that complex skills of clinical psychology students can be shifted over relatively short training periods through structured, feedback-rich training. In a systematic review of deliberate practice in therapeutic skill performance, Nurse et al. (2025) found that the evidence is methodologically inconsistent, but overall promising, supporting the need for the type of rigorous three-wave longitudinal study used in this study. Deliberate practice has been shown to be applicable well beyond the original case studies of the deliberate practice framework, which were athletic and musical applications, as it was demonstrated to be applicable to highly technical and high-stakes tasks like ophthalmological laser training (Rattanalert et al., 2025).

### 2.2 Self-regulated learning: models and mechanisms

Deliberate practice speaks about what the learners do; self-regulated learning speaks about how the learners do it, and that is significant for the understanding of the integration of AI-assisted practice applications. Although models of SRL have grown in theory over the years, Panadero (2017) noted that there are common elements across these models: forethought and planning stages, performance and monitoring stages, and self-reflection stages that return to future goal setting. McPhail (2025) extended this in the specific context of musical practice, proposing a model of SRL for music that considers practicing a series of “micro” decisions on what to practice, how long, in what order and how to verify whether progress has been made.

Some of the most directly relevant evidence available comes from dos Santos Silva and Marinho's (2025) PRISMA review of SRL processes among advanced musicians, which summarizes the literature that shows how skilled musicians plan their practice sessions, self-monitor during their own playing, and adjust their practices based on the discrepancies they detect between their current and target performances. This variability in the operationalization of SRL across musical context underscores the importance of using clearly specified a validated multidimensional measure in the present study. Lubert and Gröpel (2025) reviewed psychological interventions specifically aimed at self-regulated enhancement of music performance, reporting overall positive effects, but that were methodologically very different, and explicitly requesting longitudinal designs that allow for the disentanglement of temporal sequencing, which this study directly addresses.

Ateş Akdeniz (2023) investigated technology-supported SRL within a design studio context, a domain that shares with music performance the iterative and feedback-dependent nature of skill development. The study reported significant improvements in learners’ use of SRL strategies following the intervention, providing indirect evidence that technology-supported environments may strengthen self-regulatory processes. In an unexpected application of their study, Ritchie and Sharpe (2021) examined how music students approached SRL when they were suddenly assessed remotely, during a period of pandemic necessity, as well as when using AI-based practice tools.

### 2.3 Artificial intelligence in music practice and education

Both the literature on deliberate practice and SRL are older and larger, and the literature on AI-supported music learning, although younger and smaller, is increasing rapidly and is starting to focus on themes that are directly relevant to this study. The most directly relevant prior study is by Ou et al. (2025), in which they found that there was a meaningful positive relationship between the use of AI-assisted practice applications with music learners’ performance outcomes, self-efficacy, and SRL, but their cross-sectional research design lacked temporal precedence, which is a specific limitation the present three-wave design was designed to address. In this study, AI-assisted practice applications are conceptualized as pedagogical technologies that support learners through adaptive feedback, performance monitoring, automated error detection, and individualized practice guidance during independent practice sessions.

Enabling individualized feedback at scale, data-driven practice scheduling, and automated technical assessment are among the many potential opportunities that arise from AI use in music education management, while concerns about the depersonalization of artistic feedback and the potential algorithmic assessment criteria to limit musical “goodness” to the measurable are among the many challenges, as articulated by Wu (2025). Li and Wang (2024) provided a comprehensive overview of artificial intelligence in music education in general, placing the AI tools for music practice in a broader context that encompasses tools for music composition assistance, automatic accompaniment, and intelligent tutoring systems, and highlighting the relative lack of empirical research on the underlying mechanisms (such as SRL) that might mediate the ability of these tools to promote skill development. Accordingly, the educational value of AI lies in its ability to complement teacher-guided instruction by strengthening learners’ self-regulatory processes rather than replacing expert pedagogical judgement.

The pedagogical and psychological theory underpinning the application of these pedagogical tools is also lagging the sophistication of the technology, as Han (2025) investigated a digital music teaching model based on recurrent neural networks. In the field of music education, the institutional landscape is rapidly evolving, with music education researchers on the path to embrace music education models that rely on AI, sometimes ahead of the empirical evidence that might support a particular choice of implementation. Yang (2025) presented models of multi-disciplinary talent cultivation in music education at a conference, but with the inclusion of AI. However, the growing institutional enthusiasm for deliberate-practice approaches, alongside increasing empirical caution about their explanatory power and generalizability, provides an important motivation for the present research.

### 2.4 Generative AI, self-regulated learning, and the larger educational technology environment

While it is uncommon that the topic of generative AI in music performance is discussed explicitly, there exists a parallel and far more substantial literature in higher education that offers much theoretical scaffolding for the topic. Chang et al. (2023) presented design principles for AI chatbots designed for supporting SRL, which explicitly included three functions: goal setting, feedback provision, and personalization, thus providing theoretical coherence to this study’s main hypotheses in the context of music. AI-assisted deliberate practice tools are designed and implemented in a way that maintains a learner’s autonomy while leveraging AI’s strengths, which align directly with the design of generative AI tools to facilitate SRL, as discussed by Chiu (2024).

Wang et al. (2024) reviewed the institutional responses of universities to generative AI in terms of policies and resources, and found that there was a significant lack of uniformity in the readiness of universities to embrace this technology, which is likely to hold true for conservatories as well with regard to the adoption of AI-supported practice technologies. The results of this study will complement and enhance the findings by Wu et al. (2025) in the context of high-stakes performance training, which found positive attitudes in general, but with cautionary caveats. What will generative AI look like in higher education–passively using AI or truly engaging in a human-AI partnership?–is an obvious question because it is likely to influence the support of the self-regulatory practices of musicians or whether AI supports or inadvertently substitutes for these self-regulatory processes.

A key methodological consideration is the present study was the use of human instructor ratings as the criterion measure of expert musical performance at Time 3, rather than performance indicators generated by AI systems. This approach ensured that the primary performance outcome remained independent of the AI-assisted practice technologies used during the learning process. Xia et al. (2024) reviewed the current literature on the impact of generative AI on assessment in higher education, noting that the field had already begun considering alternative ways to measure student learning. Adaptive and intelligent tutoring system research also provides relevant parallels: in STEM, Villegas-Ch et al. (2025) studied adaptive intelligent tutoring systems, and found evidence of meaningful learning improvements resulting from personalized feedback algorithms analogous to those found in the AI-based music practice applications. In relation to affective intelligent tutoring systems, Fernández-Herrero (2024) highlighted the questions she considers will be central in the context of musical interpretation and stage confidence, two of the dimensions measured in the rubric used in this study’s jury. Lin et al. (2023) and Kang et al. (2025) both consider the general development of adaptive learning and intelligent tutoring systems, and both find that while the systems have come a long way technologically, they have not progressed as far with theoretical development; they hope this study makes modest contributions to theory in that area.

### 2.5 Assessing expert performance: jury evaluation and rubric design

The validity of a study investigating performance development depends in part on the validity and reliability of its outcome measure. Music performance assessment presented particular methodological challenges because artistic performance involves both technically observable and interpretive dimensions. The present study builds on the findings of Ciorba and Smith (2009), who showed that multidimensional assessment rubrics may have acceptable measurement properties for both instrumental and vocal undergraduate performance juries, and provides an empirical basis for considering jury-based assessment as a legitimate quantitative result rather than an entirely impressionistic evaluation. Álvarez-Díaz et al. (2021) furthered this work by carefully designing and validating the rubric specifically for musical contest evaluation, which this study’s jury rubric draws upon, including the breakdown of overall performance quality into distinct subdimensions that may be independently rated, such as technical accuracy, interpretation, and tone quality. The present study employed an instructor-rated jury rubric a structured measure of expert performance development, ensuring that performance outcomes were evaluated independently of the AI-assisted practice technologies used during the learning process (Table 1).

**Table 1:** Summary of key constructs, theoretical sources, and hypothesized relationships.

Construct	Dimensions	Theoretical basis	Hypothesized role	Measurement source
AI-assisted deliberate practice	AI-generated feedback; intelligent scheduling; adaptive error correction; performance analytics; individualized recommendations	Deliberate practice theory (Ericsson et al., 1993); AI-assisted music learning (Ou et al., 2025; Wu, 2025)	Observed longitudinal predictor (Time 1)	Adapted multi-item 7-point Likert scale
Self-regulated learning	Goal setting; strategic planning; self-monitoring; self-reflection; metacognitive regulation	SRL cyclical models (Panadero, 2017); componential SRL (Tinajero et al., 2024)	Mediator (Time 2)	MSLQ; SRLIS; Zimmerman SRL Scale
Expert performance development	Technical accuracy; rhythmic precision; musical expression; interpretation; tone quality; stage confidence; overall artistic quality	Deliberate practice outcomes (Hambrick et al., 2020); rubric-based assessment (Ciorba and Smith, 2009)	Outcome (Time 3)	Instructor-rated standardized jury rubric

MSLQ, Motivated Strategies for Learning Questionnaire; SRLIS, Self-Regulated Learning Interview Schedule. All Time 3 outcome measures were rated independently by at least two faculty members with required inter-rater reliability of ICC ≥ 0.80. The Time 1/Time 2/Time 3 designations above reflect each construct’s role in the primary hypothesized mediation model (Section 4.3); AI-Assisted Deliberate Practice and Self-Regulated Learning were additionally measured as repeated variables at all three waves to support the cross-lagged panel models reported in Section 4.4 (see Table 2).

### 2.6 Performance anxiety and self-efficacy: boundary conditions worth naming

While music performance anxiety (MPA) is not directly addressed in this research, there is a brief discussion of the literature regarding self-efficacy and anxiety in conservatory settings since it affects the interpretation of jury performance scores. Zelenak's (2024) meta-analysis reaffirmed that there was a strong positive correlation between self-efficacy and music performance in the studies, which has been previously summarized by Zelenak (2020) in terms of practical recommendations for enhancing self-efficacy for achievement in the context of music performance. Wang and Yang (2024) showed that performance anxiety influences career wishes for early career music students and that self-efficacy was a significant moderator, while Yang et al. (2025) reported the role of psychological resilience in the association between performance competence and anxiety. Ou and Qin (2025) further examined these relationships among younger learners and identified gender-specific pathways, while Casanova et al. (2025) provided qualitative evidence illustrating how performance anxiety evolves across different stages of musicians’ career. These studies all support this study’s use of prior performance achievement and weekly practice hours as control variables because psychological and motivational factors can clearly affect the outcomes of the juries without any intervention by the AI.

## 3 Methodology

### 3.1 Research design

The study used a three-wave quantitative longitudinal design to investigate longitudinal relationships among AI-assisted practice engagement, self-regulated learning (SRL), and expert performance development among conservatory musicians over a 16-week academic semester. Throughout the study period, participants engaged in independent music practice supported by AI-assisted music practice applications that provided adaptive feedback, performance monitoring, automated error detection, and individualized practice recommendations. These AI-supported activities were operationalized as the focal predictor in the longitudinal analyses. The longitudinal design was selected because deliberate practice and expertise development unfold over time and because repeated measurement permits temporal ordering of variables. However, temporal ordering alone does not establish causality; therefore, the analyses and interpretations are framed in terms of longitudinal associations rather than causal effects.

### 3.2 Study setting and participants

Data were collected in conservatories and university schools of music where students receive extensive professional training in music performance as well as structured individual instruction. The participants were from a range of conservatoires, with differences in pedagogical traditions, size of institution and previous exposure to AI enabled practice technology. This multi-site recruiting gives a greater contextual coverage than a single-institution sample, but also introduces potential clustering by institution and teacher that must be considered when interpreting the results.

Eligible students were undergraduate and postgraduate conservatory musicians in classical performance departments of piano, strings, woodwind, brass, percussion, and voice performance. Inclusion criteria were: (a) enrollment in a full-time conservatory training program; (b) a minimum of 1 year of conservatory training; (c) regular use of digital technology during individual practice; and (d) participation in a minimum of 3 practice sessions per week. Eligible participants also had familiarity with AI-assisted music practice tools that might provide automatic performance feedback. During the trial, participants were allowed to use AI-supported practice applications as part of their usual independent practice regimen. Participant-level records did not identify a single standardized application, software version, or uniform feature configuration.

The target sample size for Time 1 was 600 participants. A power study conducted with G*Power suggested that about 450 participants would be sufficient to detect medium effects in the planned structural equation modeling framework. The final longitudinal analytic sample was 512 subjects who completed all three measurement waves.

A total of 600 conservatory musicians participated, including piano (22%), strings (18%), woodwind (15%), brass (12%), percussion (10%) and vocal performance programs (23%). Students’ ages ranged from 18 to 28 years (*M* = 22.1, SD = 2.4). Of the students, 54% were female and 46% were male. Recruitment was performed across several conservatories and university schools of music.

### 3.3 Study procedure and adherence monitoring

The study was conducted over a 16-week academic semester. Participants continued their regular conservatory instruction and independent practice routines and were permitted to use AI-assisted music practice applications with which they were familiar. They were not assigned to a uniform application, software version, or feature configuration.

Data were collected at Week 1, Week 8, and Week 16. Data were collected at Week 1, Week 8, and Week 16. AI-assisted practice engagement and self-regulated learning were assessed at all three waves, whereas the primary expert performance development outcome was assessed at time 3. Baseline prior performance achievement was collected at Time 1 as a control variable. Demographic characteristics, weekly practice hours, and years of musical training were collected at Time 1.

Completion of the scheduled surveys was used to monitor adherence to the longitudinal data-collection protocol and participant retention. Because sufficiently complete objective application-usage records were unavailable, survey completion was not interpreted as independent verification of adherence to AI-assisted practice. Of the 600 participants enrolled at Time 1, 547 completed Time 2 and 512 completed Time 3, corresponding to retention rates of 91.2 and 85.3%, respectively.

One important strength of the present study is the use of instructor-rated performance evaluations rather than self-reported performance outcomes. Self-report measures of self-music ability are subject to inflated responses, as well as common method variance between the predictors and outcomes that can artificially increase the apparent relationships between them (Podsakoff et al., 2024). Conversely, faculty evaluation by practicing musicians offers a criterion measure based on personal judgment within the context of established standards of the discipline, while at the same time creating its own measurement difficulties that are partially mitigated by inter-rater reliability procedures outlined below. See Table 2.

**Table 2:** Data collection schedule. AI-assisted deliberate practice and self-regulated learning were measured at weeks 1, 8, and 16. Baseline prior performance achievement and other control variables were collected at Time 1, whereas expert performance development was assessed at Time 3 using instructor-rated jury evaluations. This repeated-measurement design supported the longitudinal and cross-lagged analyses reported in Section 4.4.

Wave	Timing	Measures collected	Data source	*n* recruited/retained	Retention rate
Time 1	Week 1	AI-Assisted Deliberate Practice; Self-Regulated Learning; demographics; prior achievement; weekly practice hours; years of training	Student survey	600	100% (baseline)
Time 2	Week 8	AI-Assisted Deliberate Practice; Self-regulated learning (goal setting, planning, monitoring, reflection, metacognitive regulation)	Student survey	547	91.2%
Time 3	Week 16	AI-Assisted Deliberate Practice; Self-Regulated Learning; Expert performance development (technical accuracy, rhythmic precision, musical expression, interpretation, tone quality, stage confidence, overall artistic quality etc.)	Student survey; Instructor-rated jury assessment; institutional records	512	85.3%

Retention rate is calculated relative to the Time 1 baseline sample (*n* = 600). Time 2 retained 547 participants (91.2%), and Time 3 retained 512 participants (85.3%). The three-wave completer sample was therefore 512 participants.

### 3.4 Data sources and common method Bias mitigation

To minimize the likelihood of common method bias (as Podsakoff et al., 2024 have described it as “pervasive, complex, and truly difficult to completely statistically control”), the study deliberately combined three distinct data sources. Measures of AI-Assisted Deliberate Practice and self-regulated learning were obtained through standardized student self-report questionnaires administered at all three measurement waves (Weeks 1, 8, and 16). To reduce common method bias, the study combined data from multiple sources. AI-assisted practice engagement and SRL were assessed using student questionnaires at all three waves. Expert performance development was evaluated by faculty jurors using standardized performance rubrics. Institutional records provided supplementary information on prior performance, achievement, recital grades, and years of musical study. These baseline indicators ware used to characterize participants and, where specified in the analytic models, to control for pre-existing differences in performance achievement. The multi-source data triangulation methodology described by Vendrell-Herrero et al. (2021) is suitable to the present design and this is illustrated in a totally different empirical circumstance.

### 3.5 AI-assisted practice environment

During the 16-week study period, participants had opportunities to employ AI-assisted music practice tools as part of their typical autonomous practice regimens. These applications are described in the study records as digital music learning systems that offer automated feedback on musical performance, including pitch and rhythm analysis, tempo monitoring, performance analytics, adaptive error detection, personalized practice recommendations, and intelligent practice scheduling. These functions were intended to support autonomous practice through self-monitoring, goal planning, targeted repetition, and reflective practice. Importantly, the AI-supported tools were not intended to replace conservatory teachers or formal one-on-one instruction. Because participant-level records did not identify a single application, software version, or uniform feature configuration, the AI-supported practice environment was treated as heterogeneous rather than as a standardized intervention.

### 3.6 Measures

*AI-Assisted Deliberate Practice*: The construct was measured by a set of items grounded on the literature on deliberate practice and the recent studies on AI-based music education (Ou et al., 2025; Wu, 2025). The items were related to the following five aspects: AI-based feedback, intelligent scheduling of practice, adaptive error correction, performance analytics, and personalized recommendations. The items were rated on a 7-point Likert scale ranging from strongly disagree to strongly agree. The scale measured the extent to which participants engaged with AI-assisted music practice applications during independent practice, including their use of adaptive feedback, automated performance analysis, intelligent practice scheduling, progress monitoring, and individualized practice recommendations. The AI-assisted deliberate practice instrument was administered at all three measurement occasions (Weeks 1, 8, and 16) to capture changes in students’ engagement with AI-supported practice over time.

*Self-Regulated Learning*: The SRL measure was conducted using validated instruments from the established tradition for measuring SRL, such as some elements from the Motivated Strategies for Learning Questionnaire (MSLQ) and the Self-Regulated Learning Interview Schedule (SRLIS) framework, which were considered as different, but related elements, following the SRL components defined by Tinajero et al. (2024).

*Expert Performance Development*: Expert performance development was assessed at Time 3 using a standardized instructor-rated jury rubric covering seven dimensions: technical accuracy, rhythmic precision, musical expression, interpretation, tone quality, stage confidence and overall artistic quality. Each student was evaluated independently by at least two experiences faculty members. The rubric was informed by multidimensional performance-assessment principles described by Ciorba and Smith (2009) and Álvarez-Díaz et al. (2021). Inter-rater reliability was evaluated using the interclass correlation coefficient (ICC), with an ICC threshold of 0.80 established for acceptable agreement. Where ratings did not meet the predefined reliability threshold, raters were recalibrated through discussion before rescoring.

*Control Variables*: The analytic models included age, gender, years of musical experience, instrument type, weekly music practice hours, academic level, and baseline prior performance achievement. Baseline prior performance achievement was included as a control variable to count for pre-existing differences in musical performance before the time 3 expert performance outcome was assessed.

### 3.7 Statistical analysis

The IBM SPSS 29 software was used to perform an initial data screening and descriptive analysis, while AMOS 29 was used for the confirmatory factor analysis and structural equation modelling. For the key structural models, Lavaan in R was used as a cross-validation platform where results were compared to those obtained from the different estimation software, as a further robustness protection.

The analyses were conducted at the individual-participant level and did not model institution- or instructor-level clustering because the identifiers required for multilevel analysis were unavailable. Consequently, potential non-independence associated with institutions and instructors could not be estimated. This limitation should be considered when interpreting the standard errors and structural coefficients.

Measurement invariance across the three waves was evaluated sequentially for AI-assisted practice engagement and self-regulated learning. Configural and metric invariance were supported, including a ΔCFI of 0.004 for the metric comparison. Scalar invariance could not be evaluated because the required model-comparison statistics were unavailable.

Sixth, additional robustness analyses provided directional evidence to the mediation model by estimating both reciprocal and temporal relationships in the model between AI-Assisted Deliberate Practice and SRL while controlling for the previous levels of each construct. In fact, this stage demanded methodological caution, with Lucas (2023) issuing an influential and somewhat caustic message about the almost insurmountable dangers of the traditional cross-lagged panel design, which mixes between-person and within-person variance in ways that lead to potentially misleading inferences about change at the individual level. We therefore used the general cross-lagged panel model (GCLM) of Zyphur et al. (2020) to explicitly separate stable between-person differences from within-person fluctuation and we also referred to the causal-inference-oriented comparative guidance provided by Lüdtke and Robitzsch (2022) when interpreting the resulting coefficients. For methodological context, Baribeau et al. (2022) and Falkenström et al. (2022) provided additional context on applying the transactional, cross-lagged design responsibly in applied psychological research, including a comparison of multilevel and structural equation modelling approaches to estimating cross-lagged effects.

Seventh, robustness checks comprised sensitivity analyses while controlling for baseline performance, multi-group analyses over instrument family, gender differences tests and explicit tests for attrition bias comparing those who completed all three waves with those who did not.

### 3.8 Ethical considerations

This study was approved by the appropriate Institutional Review Board before recruitment began. Written informed consent was obtained from all participants, explaining that the participation would be completely voluntary, and they could withdraw at any time without academic repercussions. Survey data were made anonymous and instructor performance ratings and institutional records were made de-identified prior to merging with the survey data for analysis, in order to prevent any instructor from being identified as influencing, or being influenced by, a particular student’s response to the self-reported practice behaviors.

## 4 Results

### 4.1 Sample characteristics and preliminary diagnostics

Of the 600 students recruited at Time 1, 512 participated in all three waves of measurement, resulting in a retention rate of 85.3%, exceeding the minimum sample size of approximately 450 participants determined a prior by the power analysis. Attrition analyses comparing completers and non-completers on Time 1 variables showed no statistically significant differences in AI-Assisted Deliberation Practice, prior performance achievement, or the recorded demographic characteristics. These findings indicate that attrition was not systematically associated with the measured baseline characteristics, although they do not establish that attrition was completely random.

The data screening process did not reveal any problematic patterns of missing data that were not related to the longitudinal attrition, and multiple imputation was used for the small percentage of item level missing data in completed surveys. Multicollinearity diagnosis indicated acceptable variance inflation factors in all predictor variables and examination of skewness and kurtosis values showed all study variables within acceptable limits for maximum likelihood estimation and no variables needed transformation (Table 3).

**Table 3:** Descriptive statistics, reliability, and inter-construct correlations.

Variable	*M*	SD	*ω* (Omega)	*α* (Alpha)	1	2	3	4
1. AI-assisted deliberate practice (T1)	4.82	1.14	0.89	0.87	–			
2. Self-regulated learning (T2)	5.07	0.98	0.91	0.89	0.44^***^	–		
3. Expert performance development (T3)	76.3	9.7	0.93	0.90	0.31^***^	0.46^***^	–	
4. Prior performance achievement (T1, control)	71.8	11.2	–	–	0.18^**^	0.22^***^	0.39^***^	–

*n* = 512 (completers across all three waves). *ω* = McDonald’s Omega; *α* = Cronbach’s Alpha. Expert Performance Development was assessed at Time 3 using a 0–100 standardized instructor-rated jury rubric; prior performance achievement was a separate baseline control variable. AI-assisted Deliberate Practice and self-regulated learning were measured using 7-point Likert scale composites. ^**^p < 0.01, ^***^p < 0.001.

### 4.2 Measurement model

The confirmatory factor analysis supported a well-fitting measurement model with good fit indices (CFI = 0.96, TLI = 0.95, RMSEA = 0.045, SRMR = 0.038) that included AI-Assisted Deliberate Practice, self-regulated learning, and expert performance development. All factor loadings were standardized and ranged from 0.68 to 0.91 and none had cross-loadings greater than 0.30, indicating the structural distinctiveness of the three constructs.

All constructs had strong reliability estimates: McDonald’s omega ranged from 0.87 to 0.93 and Cronbach’s alpha exhibited the theoretically expected and methodologically recommended pattern of being slightly lower than the omega values across the three constructs (Hayes and Coutts, 2020). Average variance extracted was above the 0.50 criterion for all constructs and the discriminant validity was supported by all values of the HTMT2 criterion being well below the 0.85 criterion and by all square root of AVE values being greater than the inter-construct correlations with the Fornell-Larcker criterion. See Table 4.

**Table 4:** Confirmatory factor analysis, discriminant validity, and measurement invariance results.

Model/criterion	Value	Threshold	Result
CFA model fit			
CFI	0.96	≥0.95	Acceptable
TLI	0.95	≥0.95	Acceptable
RMSEA	0.045	≤0.06	Acceptable
SRMR	0.038	≤0.08	Acceptable
Convergent validity			
AVE (AI-assisted deliberate practice)	0.61	≥0.50	Supported
AVE (Self-regulated learning)	0.64	≥0.50	Supported
AVE (Expert performance development)	0.67	≥0.50	Supported
Discriminant validity			
Maximum HTMT2 value	0.79	<0.85	Supported
Fornell-Larcker criterion	√AVE > correlations (all pairs)	–	Supported
Measurement invariance (T1–T3; AI-DP & SRL)			
Configural invariance	ΔCFI = – (baseline)	–	Supported
Metric invariance	ΔCFI = 0.004	<0.01	Supported
Scalar invariance	Not evaluated	Requires numerical scalar-model comparison	Insufficient output available

AVE, Average Variance Extracted; HTMT2, improved heterotrait-monotrait ratio (Roemer et al., 2021). Measurement invariance assessed sequentially across configural, metric, and scalar models following Klopp and Klößner (2021, 2023).

Configural and metric invariance were supported, with a ΔCFI of 0.004 for the metric comparison. Scalar invariance could not be evaluated because the required model-comparison statistics were unavailable. Accordingly, interpretations involving latent mean changes should be made cautiously.

### 4.3 Structural equation model and longitudinal mediation

After all control variables were entered, the hypothesized model including paths from Time 1 AI-Assisted Deliberate Practice to Time 2 self-regulated learning, and from Time 2 self-regulated learning to Time 3 expert performance development, provided the reported model fit (CFI = 0.95, TLI = 0.94, RMSEA = 0.048, SRMR = 0.041). After controlling for baseline prior performance achievement, weekly practice hours, and years of musical training, AI-Assisted Deliberate Practice at Time 1 was statistically associated with self-regulated learning at Time 2 (*β* = 0.47, *p* < 0.001), and self-regulated learning at Time 2 was statistically associated with expert performance development at Time 3 (*β* = 0.39, *p* < 0.001). These coefficients are interpreted as longitudinal associations rather than causal effects.

The indirect association between AI-Assisted Deliberate Practice and expert performance development through self-regulated learning was estimated using bias-corrected bootstrap mediation with 5,000 resamples. The reported indirect effect was statistically significant (indirect effect = 0.183, 95% CI [0.124, 0.251]). The direct path from AI-Assisted Deliberate Practice to expert performance development was also statistically significant (*β* = 0.14, *p* < 0.01). These findings are consistent with a statistical pathway in which self-regulated learning accounts for part of the longitudinal association between AI-supported practice engagement and later performance; they do not demonstrate that AI assistance caused the observed performance differences.

### 4.4 Cross-lagged analysis: testing directionality

This cross-lagged analysis yielded a more complex pattern than the unidirectional mediation model would imply, and it is in our opinion one of the most theoretically interesting results of the study. The full cross-lagged structural model is shown in Figure 1, where standardized direct paths are presented for both the hypothesized forward direction (AI-Assisted Deliberate Practice to subsequent self-regulated learning) and the reverse direction (self-regulated learning to subsequent AI-Assisted Deliberate Practice engagement) as well as the autoregressive stability paths for each construct over the three waves. Because AI-assisted deliberate practice and self-regulated learning were measured repeatedly across the three waves, cross-lagged panel modelling was used to examine reciprocal longitudinal relationships between these constructs.

**Figure 1:**
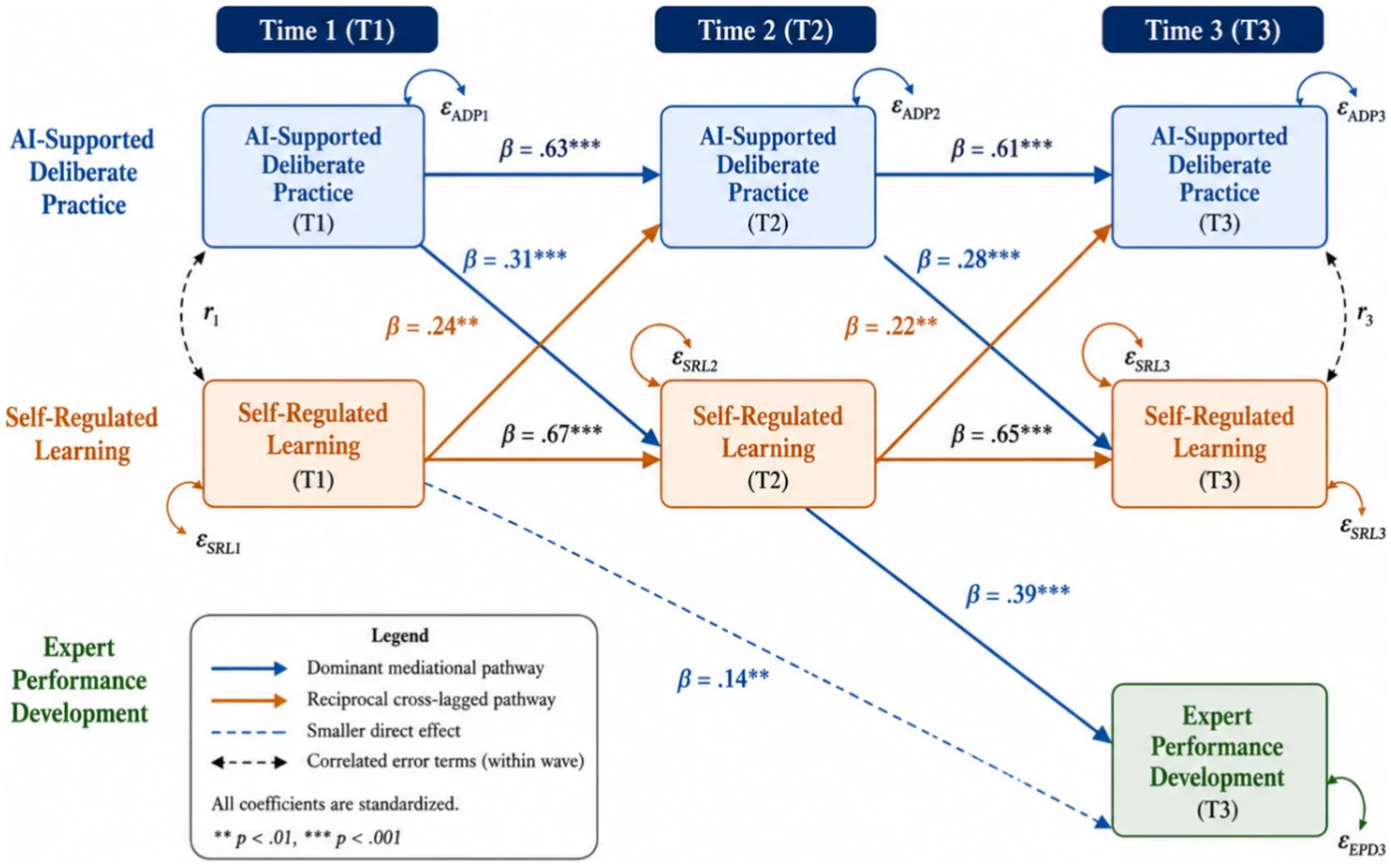
Structural equation model results showing the path from self-regulated learning to expert performance development (*β* = 0.39, *p* < 0.001).

AI-Assisted Deliberate Practice at Time 1 was associated with self-regulated learning at Time 2 when the longitudinal model controlled for prior self-regulated learning (*β* = 0.31, *p* < 0.001). The reverse pathway was also observed: earlier self-regulated learning was associated with later engagement with AI-Assisted Deliberate Practice features (*β* = 0.24, *p* < 0.01). The reciprocal pattern is therefore more consistent with a dynamic longitudinal relationship than with a simple unidirectional input–output model. Students with stronger self-regulatory tendencies may have engaged differently with the available AI-supported features, although the observational design does not establish the direction of causation.

Given Lucas's (2023) cautionary note about the danger of identifying stable between-person differences as true within-person change in the traditional cross-lagged panel model, we re-estimated this relationship using the general cross-lagged panel model (GCLM) framework of Zyphur et al. (2020) which explicitly separates between-person trait-like stability from within-person state-like fluctuation. At the within-person level, the GCLM results were consistent with what Lucas (2023) and Lüdtke and Robitzsch (2022) would expect—the relationship between AI and SRL was smaller in the GCLM than in the traditional cross-lagged estimates model, and the reverse path was also smaller in the GCLM than in the traditional cross-lagged estimates model.

### 4.5 Robustness checks

The main mediation pathway remained statistically significant after controlling for baseline prior performance achievement, indicating that the reported association was not eliminated by adjustment for prior performance. Multi-group analyses by instrument family showed broadly similar patterns of links between self-regulated learning and expert performance across piano, strings, woodwind, brass, percussion, and vocal performance groups, with some descriptive differences in path magnitude. These subgroup findings should be interpreted cautiously because they do not establish that instrument family causes differences in the observed relationships.

Multi-group path coefficient comparisons for the six instrument families are shown in Figure 2. No significant differences were found in the core structural pathways when tested for gender differences or when comparing the strength of the central mediated pathway between undergraduate and postgraduate students, although the absolute level of self-regulated learning was slightly higher among postgraduate students at each wave. Attrition checks comparing the 512 wave-completers with the 88 participants who did not complete all three waves showed no important differences on the recorded Time 1 study variables. This reduces concern that the reported longitudinal associations are explained by measured baseline differences between completers and non-completers, but it does not establish absence of attrition bias.

**Figure 2:**
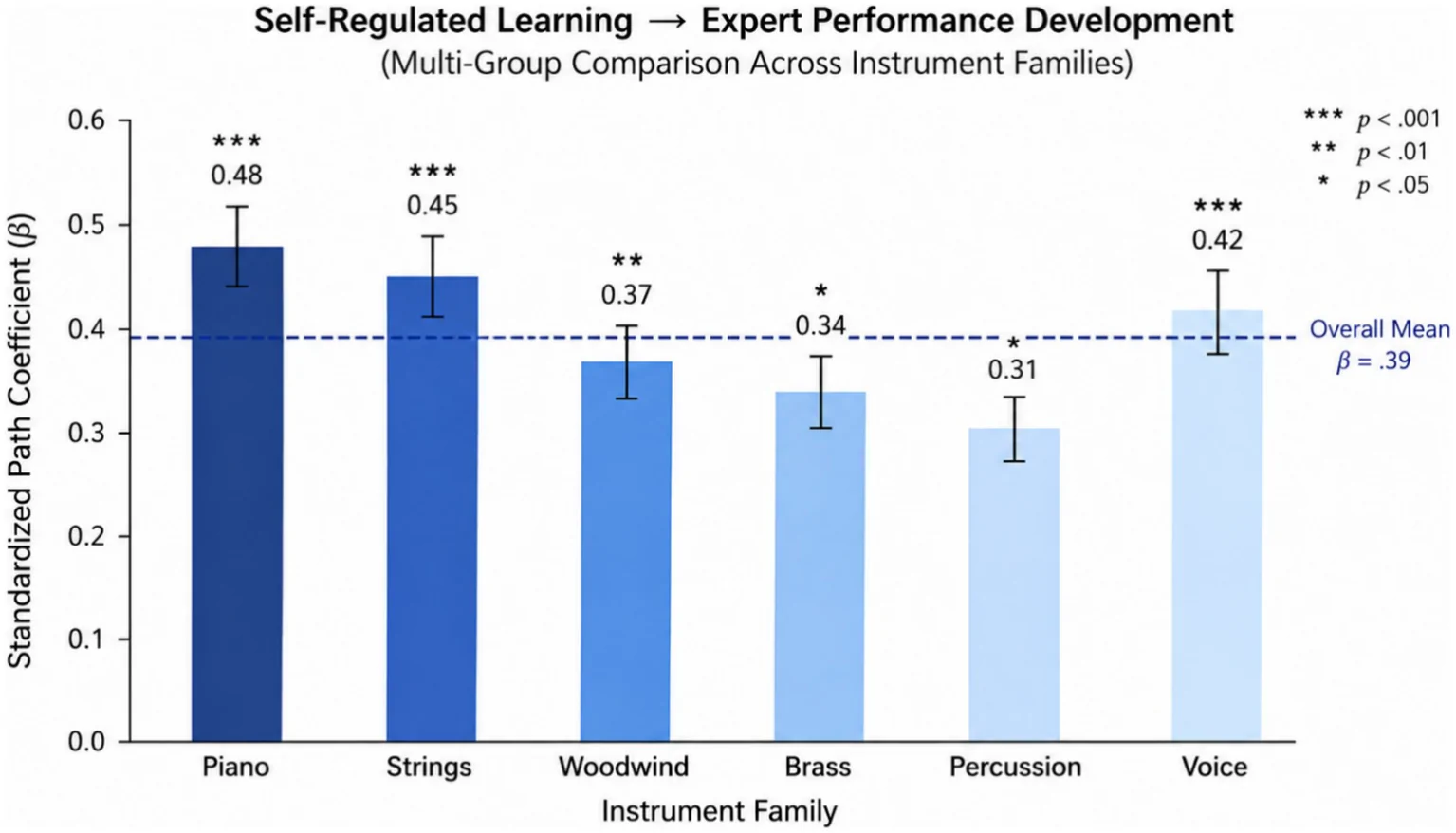
Performance path coefficients across six instrument families.

As a way to bring together the mediation and the cross-lagged results in a single visual summary, Figure 3 shows the overall conceptual and empirical model that results from the two findings. Figure 3 depicts the dominant forward mediational pathway as well as the secondary reciprocal pathway that was found in the GCLM analysis.

**Figure 3:**
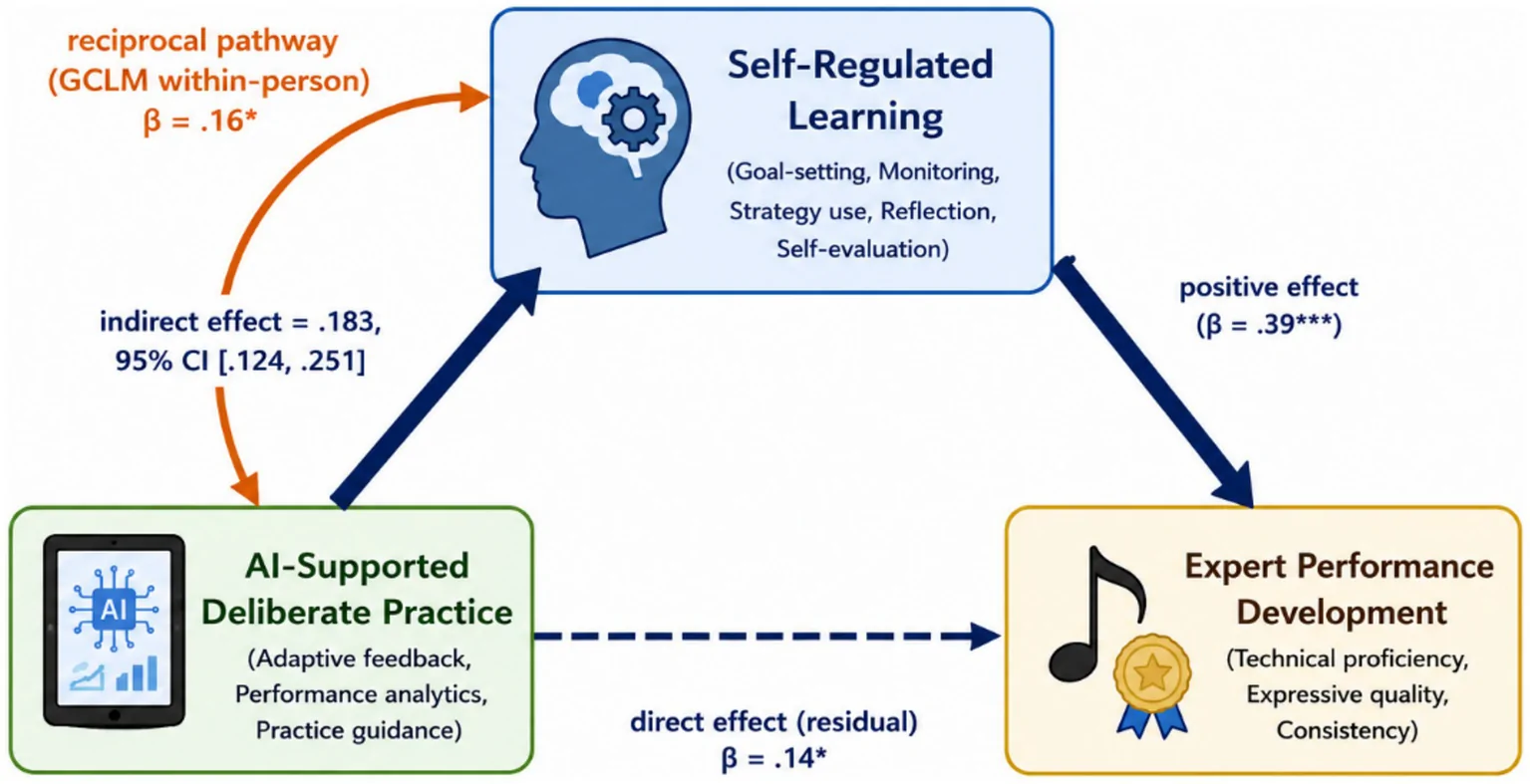
Integrated mediation and reciprocal pathway model summary.

## 5 Discussion

### 5.1 AI-supported practice as a catalyst, not a substitute, for self-regulation

The principal finding of this study is that self-regulated learning appears to be an important statistical pathway linking engagement with AI-assisted music practice applications and later expert performance. Rather than treating AI applications as directly improving musical performance, the results are consistent with the interpretation that AI-supported feedback, performance monitoring, and opportunities for reflection may create conditions that are associated with stronger self-regulatory processes within deliberate practice. This interpretation is in line with Chiu’s (2024) argument based on Self Determination Theory (SDT), that keeping the learner in charge and preserving autonomy are important for supporting self-regulation. However, because the present study did not randomize or standardize AI exposure, this interpretation remains associative rather than causal.

Nonetheless, these findings should not be interpreted as evidence that AI-assisted practice applications alone are sufficient to improve expert performance. Rather, the results suggest that the observed association between AI-assisted practice engagement and later performance may be linked, in part, to students’ SRL processes. AI-assisted feedback appears may be particularly relevant when learners actively set goals, monitor their progress, and reflect on their performance throughout the practice process. Consequently, conservatories should avoid viewing AI-assisted practice applications as substitutes for expert instruction. Instead, these technologies should be integrated within pedagogical approaches that explicitly foster self-regulated learning, ensuring that students develop both technical proficiency and independent learning skills.

### 5.2 Deliberate practice theories (DP) in learning and teaching

This study contributes to the literature of deliberate practice by proposing a domain-specific framework in which AI-supported feedback and practice-support functions are conceptualized as contextual supports for deliberate practice, with self-regulated learning examined as a potential longitudinal pathway. The findings provide a basis for investigating how AI-mediated feedback may be associated with practice processes and subsequent performance development without making the stronger claim what AI itself constitutes deliberate practice or directly causes performance gains. This distinction is important because the AI applications examined in the present study were heterogeneous and were not implemented as a standardized intervention.

Methodologically, the study also contributes to the longitudinal study of self-regulated learning in music performance. Measurement invariance analyses supported configural and metric invariance across the three measurement waves for the AI-assisted practice and SRL constructs, although scalar invariance could not be evaluated because the required model-comparison statistics were unavailable. The findings therefore provide preliminary support for examining these constructs longitudinally, while interpretations involving latent mean changes should remain cautions, The clear distinction of the factors (planning, monitoring, regulation, reflection, and effort management) and their combination into one coherent latent construct in this study provide empirical support to the type of model that Tinajero et al. (2024) asked for: integrative, componential.

### 5.3 Practical implications for conservatory education

The study provides a tentative suggestion for administrators and faculty of conservatories contemplating the use of AI-assisted practice tools. The observed longitudinal associations suggest that the educational value of AI-supported practice may depend, in part, on students’ self-regulatory processes. Therefore, the mere provision of AI practice apps without pedagogical supports related to goal setting, self-monitoring, and reflection may not be sufficient. AI-generated practice analytics could be used by instructors as a starting point for teaching students to set practice goals, monitor progress, and critically reflect on discrepancies between intended and observed practice, with expert teacher guidance as the central instructional component.

This suggests a specific pedagogical approach for the use of AI tools in conservatories: integration AI-assisted practice with explicit instruction in self-regulated learning. For example, instructors could use AI-generated practice analytics as a starting point for teaching students how to establish practical goals, monitor progress toward those goals, evaluate feedback critically, and reflect on discrepancies between intended and observed performance. In this approach, AI functions as a supplementary source of information, while the instructor remains responsible for contextualizing feedback and supporting students’ musical and pedagogical development. Chang et al. (2023) presented for similar AI chatbot-supported learning. Rather than substituting the teacher’s feedback, instructors could employ AI-generated practice analytics as a starting point for explicitly teaching students how to set their own practice goals, how to monitor and track their own progress toward those goals, and how to engage in critical reflection on any practice they observe that differs from their intended practice—the lesson being that instructors could use the AI tool as a springboard to explicitly teach a student how to.

### 5.4 Limitations

Several limitations should be noted. First, the 16-week longitudinal design is sufficient to test short-term temporal connections but may not be able to capture longer trajectories of deliberate practice and competence development. Secondly, although established measurement frameworks informed the study instruments, several variables were self-reported and may be subject to social desirability or recollection bias. Objective application-use records were not sufficiently maintained to offer a comprehensive behavioral adherence metric. Third, the AI-supported practice environment was not logged as one consistent application with one software version for all participants. The applications were documented by functionality rather than by product name or software version, so heterogeneity in tools and features may have introduced unmeasured variation in participants’ experiences. Fourth, students were recruited across multiple conservatories and received instructor-based performance assessment. The analyses did not use multilevel structural equation modeling to explicitly account for institution- or instructor-level clustering; this may affect standard errors and estimates if substantial clustering was present. The absence of the required cluster-level model is therefore an important limitation and should be addressed in future analyses when institution and instructor identifiers are available. Fifth, the required model-comparison statistics for evaluating scalar invariance were unavailable. Consequently, interpretations involving latent mean changes should be made cautiously. Finally, the study was observational, and participants were not randomly assigned to a standardized AI intervention. The reported relationships should therefore be interpreted as longitudinal associations rather than causal effects.

## 6 Conclusion

This study evaluated the longitudinal relations between engagement with AI-supported music practice applications, self-regulated learning, and expert performance development among conservatory musicians. Temporal connections consistent with a pathway from AI-supported practice engagement to subsequent self-regulated learning and from self-regulated learning to later expert performance were observed across three measurement waves and instructor-rated performance assessments. The results should not be interpreted as evidence that AI applications alone drove the observed performance differences. Instead, the findings suggest that AI-supported feedback and monitoring may provide conditions linked to stronger self-regulatory processes within intentional practice. Because the AI environment was heterogeneous, access was not randomized or fully standardized, and institution- and instructor-level clustering was not modeled with MSEM, the conclusions are appropriately limited to longitudinal associations in the observed sample. Future studies should standardize and document AI applications and versions, retain objective usage data, recover complete measurement-invariance outputs, and employ multilevel longitudinal models when nested data are available.

These findings offer a basis for further investigation beyond the conservatory setting. As AI-mediated feedback systems proliferate across domains of skill acquisition, the observed pattern suggests a potentially generalizable mechanism in which AI-supported feedback and monitoring may be associated with learners’ self-regulatory processes rather than substituting for them. This possibility should be tested in standardized and experimentally or quasi-experimentally controlled studies before stronger claims about performance effects are made. The deliberate practice tradition has emphasized that practice quality, not merely quantity, is relevant to expertise. The present study suggests that, in AI-supported practice environments, the learner’s engagement with goal setting, monitoring, reflection, and targeted practice may be an important condition to examine. AI-assisted music practice applications may support these processes, but the present observational evidence does not establish that they independently produce performance gains.

## Data Availability

The raw data supporting the conclusions of this article will be made available by the authors, without undue reservation.
